# Retraction Note: Reversible inhibitor of CRM1 sensitizes glioblastoma cells to radiation by blocking the NF-κB signaling pathway

**DOI:** 10.1186/s12935-024-03508-w

**Published:** 2024-09-18

**Authors:** Xuejiao Liu, Yiming Tu, Yifeng Wang, Di Zhou, Yulong Chong, Lin Shi, Guanzheng Liu, Xu Zhang, Sijin Wu, Huan Li, Shangfeng Gao, Mingshan Niu, Rutong Yu

**Affiliations:** 1 Insititute of Nervous System Diseases, Affiliated Hospital of Xuzhou Medical University, Xuzhou Medical University, Xuzhou, Jiangsu China; 2 Department of Neurosurgery, Affiliated Hospital of Xuzhou Medical University, Xuzhou, Jiangsu China; 3 Department of Neurosurgery, The First Affiliated Hospital of Nanjing Medical University, Nanjing, Jiangsu China; 4 Department of Neurosurgery, Suqian Hospital Affiliated to Xuzhou Medical University, Suqian, Jiangsu China; 5 College of Pharmacy, The Ohio State University, Columbus, OH USA; 6 Blood Diseases Institute, Xuzhou Medical University, Xuzhou, Jiangsu China

**Cancer Cell International (2020) 20:97**

10.1186/s12935-020-01186-y.

**Retraction Note**

The Editor-in-Chief has retracted this article at the authors’ request. After publication, concerns were raised regarding the data included in the figures. Specifically:

- Fig. 1e 0 Gy images appear to be assembled incorrectly, and the EdU images for 0.125 and 0.25 µM S109 appear highly similar;
- Fig. 2c 0.25 µM 6 Gy image appears highly similar to Fig. 1i (C6 cells, 25 µM GDC-0449) of another article [1] from the same author group;
- Fig. 4b 0 mg/kg S109 10 Gy Day 10 (2nd mouse) and Day 18 (2nd mouse) images appear highly similar;
- Fig. 4e 0 mg/kg S109 10 Gy and 50 mg/kg S109 0 Gy images appear to overlap.

The authors have stated that incorrect images were included in the figure panels during figure preparation. Although the authors believe that these issues do not affect the conclusions of the study, they requested to retract the article to prevent any potential confusion or misinterpretation by readers.

Xuejiao Liu stated on behalf of all authors that all authors agree to this retraction.
